# Dissolution-precipitation growth of uniform and clean two dimensional transition metal dichalcogenides

**DOI:** 10.1093/nsr/nwaa115

**Published:** 2020-05-30

**Authors:** Zhengyang Cai, Yongjue Lai, Shilong Zhao, Rongjie Zhang, Junyang Tan, Simin Feng, Jingyun Zou, Lei Tang, Junhao Lin, Bilu Liu, Hui-Ming Cheng

**Affiliations:** Shenzhen Geim Graphene Center, Tsinghua−Berkeley Shenzhen Institute and Tsinghua Shenzhen International Graduate School, Tsinghua University, Shenzhen 518055, China; Shenzhen Geim Graphene Center, Tsinghua−Berkeley Shenzhen Institute and Tsinghua Shenzhen International Graduate School, Tsinghua University, Shenzhen 518055, China; Shenzhen Geim Graphene Center, Tsinghua−Berkeley Shenzhen Institute and Tsinghua Shenzhen International Graduate School, Tsinghua University, Shenzhen 518055, China; Shenzhen Geim Graphene Center, Tsinghua−Berkeley Shenzhen Institute and Tsinghua Shenzhen International Graduate School, Tsinghua University, Shenzhen 518055, China; Shenzhen Geim Graphene Center, Tsinghua−Berkeley Shenzhen Institute and Tsinghua Shenzhen International Graduate School, Tsinghua University, Shenzhen 518055, China; Shenzhen Geim Graphene Center, Tsinghua−Berkeley Shenzhen Institute and Tsinghua Shenzhen International Graduate School, Tsinghua University, Shenzhen 518055, China; Shenzhen Geim Graphene Center, Tsinghua−Berkeley Shenzhen Institute and Tsinghua Shenzhen International Graduate School, Tsinghua University, Shenzhen 518055, China; Shenzhen Geim Graphene Center, Tsinghua−Berkeley Shenzhen Institute and Tsinghua Shenzhen International Graduate School, Tsinghua University, Shenzhen 518055, China; Department of Physics, Southern University of Science and Technology, Shenzhen 518055, China; Shenzhen Geim Graphene Center, Tsinghua−Berkeley Shenzhen Institute and Tsinghua Shenzhen International Graduate School, Tsinghua University, Shenzhen 518055, China; Shenzhen Geim Graphene Center, Tsinghua−Berkeley Shenzhen Institute and Tsinghua Shenzhen International Graduate School, Tsinghua University, Shenzhen 518055, China; Shenyang National Laboratory for Materials Sciences, Institute of Metal Research, Chinese Academy of Sciences, Shenyang 110016, China

**Keywords:** dissolution-precipitation growth, two dimensional materials, transition metal dichalcogenides, uniform, clean

## Abstract

Two dimensional transition metal dichalcogenides (TMDCs) have attracted much interest and shown promise in many applications. However, it is challenging to obtain uniform TMDCs with clean surfaces, because of the difficulties in controlling the way the reactants are supplied to the reaction in the current chemical vapor deposition growth process. Here, we report a new growth approach called ‘dissolution-precipitation’ (DP) growth, where the metal sources are sealed inside glass substrates to control their feeding to the reaction. Noteworthy, the diffusion of metal source inside glass to its surface provides a uniform metal source on the glass surface, and restricts the TMDC growth to only a surface reaction while eliminating unwanted gas-phase reaction. This feature gives rise to highly uniform monolayer TMDCs with a clean surface on centimeter-scale substrates. The DP growth works well for a large variety of TMDCs and their alloys, providing a solid foundation for the controlled growth of clean TMDCs by the fine control of the metal source.

## Teaser

2D materials with good quality and ultraclean surface were obtained by a universal dissolution-precipitation growth method that precisely controls the metal source and restricts the growth of materials to surface reaction.

## INTRODUCTION

Two dimensional (2D) transition metal dichalcogenides (TMDCs) have attracted increasing attention due to their atomically thin body, excellent electronic and optoelectronic properties, and abundant material choices [1–4]. Chemical vapor deposition (CVD) is an important method of preparing TMDCs and great success has been achieved in growing large single crystals as well as continuous films [5–9]. Currently, one bottleneck in the CVD growth of TMDCs is that it is difficult to prepare uniform, large-area, and ultraclean monolayer TMDCs, because the metal sources can hardly be precisely controlled using current feeding methods [10–12]. In a typical TMDC growth process, solid sources like MoO_3_ and sulfur (S) powders are used. First, the feed amount of Mo is location dependent, which means that the Mo concentration is different at different positions on the substrate, causing a non-uniform MoS_2_ distribution [10,13]. Second, the MoO_3_ and S feeds share the same diffusion path so that there may be gas-phase reactions in addition to the on-substrate reaction, causing by-product deposited on surface of as-grown MoS_2_. There has been much effort to solve these problems, such as using liquid- or gas-phase Mo and S sources, and pre-deposition of the Mo source [14–16]. Nevertheless, it is still difficult to grow uniform and ultraclean TMDCs over large areas [17,18].

To tackle these issues, we may learn the lesson from graphene growth [19–21]. In typical graphene growth by CVD, the carbon source is dissolved in the bulk or sub-surface of catalytic substrates like nickel or copper, followed by precipitation at the substrate surface to grow uniform graphene over large areas [22,23]. This mechanism also works well for other 2D materials. For example, Shi *et al*. have used a molten Fe_82_B_18_ alloy which supplies boron source and dissociates nitrogen in the carrier gas for the growth of multilayer boron nitride [24]. Li *et al*. have recently reported that MoS_2_ ribbons can be grown by forming Na-Mo-O droplets through a vapor-liquid-solid growth mechanism [25,26]. Therefore, analogous to the mechanism for growing graphene, it is possible to grow uniform and clean 2D TMDCs, if one can ‘dissolve’ the metal source into the growth substrate to control its feed [27].

In this work, we report a ‘dissolution-precipitation’ (DP) growth method that achieves the dissolution of the required metal source into the growth substrate and succeeds in growing uniform and clean monolayer TMDCs. In this method, the metal source is embedded between two pieces of glass, and gradually diffuses out to the surface of the upper glass during growth. In this way, we have (i) achieved a uniform feed of the metal source and (ii) restricted the reaction to only the surface of the top glass while eliminated any unwanted gas-phase reactions because the metal and chalcogen sources do not share the same diffusion path [23,28]. As a result of these two features, highly uniform monolayer TMDCs with a clean surface have been grown on a centimeter-scale molten glass surface. The method has been used for many different TMDCs and their alloys, such as MoSe_2_, WS_2_, MoTe_2_, Mo_x_W_1-x_S_2_ and V doped MoS_2_, showing good universality.

## RESULTS AND DISCUSSION

**Figure 1.**
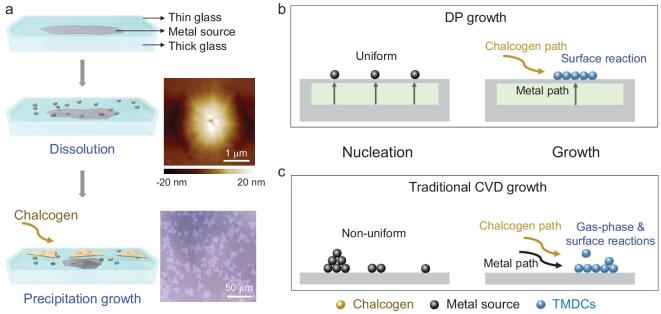
The DP growth of TMDCs in comparison with the traditional CVD method. (a) Illustration of the DP growth process. The upper inset is an AFM image of one representative protrusion on the upper thin glass surface and the bottom inset is the optical image of DP-grown MoS_2_. (b, c) Comparisons between (b) DP growth and (c) traditional CVD growth of TMDCs in the nucleation process and the growth process.

A scheme of the DP growth of TMDCs is illustrated in Fig. 1a. A metal source (e.g. Na_2_MoO_4_, Na_2_WO_4_, NaVO_3_) was embedded between two pieces of glass (the thickness of the bottom is 2 mm and the top one is 0.15 mm) to make a glass/metal source/glass sandwich structure (SG-M), which was then heated and fused together. This sandwich was used as a substrate and the metal source for the growth of TMDCs. The chalcogen source (S, Se, or Te powder) was placed at the upper steam in a horizontal tube furnace and the growth was conducted at 700–800°C depending on the material used. With increasing temperature, the metal source melted and diffused through the molten glass to its surface [28–30], which is called the ‘dissolution’ process. An AFM image of a typical surface of the top glass (Fig. 1a) shows a protrusion with a lateral size of ∼2 μm and a height of ∼20 nm, which serves as the metal source for subsequent TMDC growth. From the volume of a protrusion, we estimate that the amount of Mo precursor in each protrusion is around 1.8 × 10^−13 ^g. Such a low concentration of metal source in the feed has been effective in many CVD process [28,31,32]. After diffusing to the upper surface, the metal source reacts with the chalcogen to grow TMDCs on the molten glass surface, which is called the ‘precipitation’ process. The details of the growth mechanism are discussed in Figs S1–7. In the nucleation stage, the liquid-phase metal source uniformly diffuses to surface (Fig. 1b), guaranteeing a uniform supply. In contrast, in the traditional CVD growth of TMDCs where the metal precursor is a solid powder which sublimates in a hard-to-control manner, the metal source concentration depends on the distance from the solid source (Figs 1c and S8) [13]. In addition, during DP growth, the metal source and chalcogen vapor meet only on the substrate surface, so only here does the surface reaction happen (Fig. 1b). This is distinct from traditional CVD where the metal source and chalcogen vapor first meet in the gas phase and both gas-phase and surface reactions occur, resulting in the non-uniform nucleation and growth of TMDCs, as well as deposition of gas phase products on the TMDC surface (Fig. 1c). Therefore, this DP method grows TDMCs that are both uniform and clean compared to the traditional CVD method.

**Figure 2.**
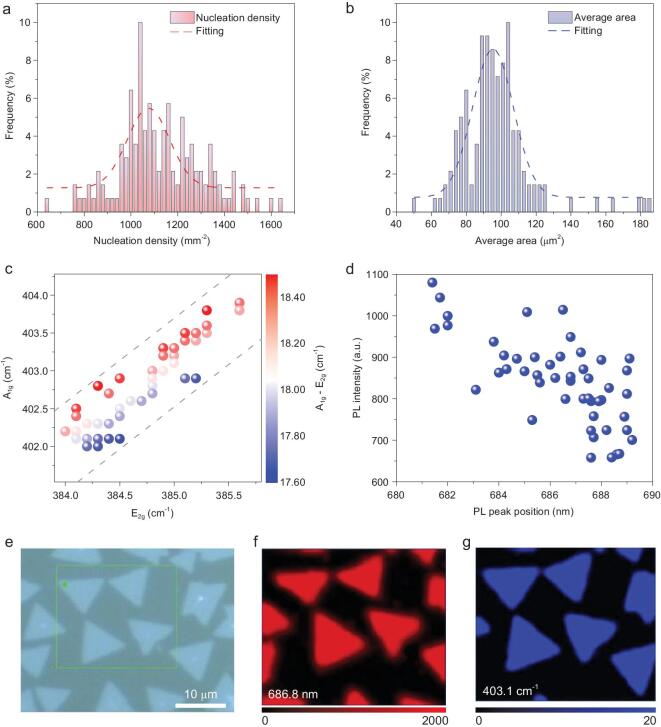
High uniformity of DP-grown monolayer MoS_2_ flakes on a 2.5 × 1.0 cm^2^ glass substrate. Statistical analysis of (a) nucleation density and (b) average area of each MoS_2_ flake from 140 images. Statistical analysis of (c) Raman peak positions and (d) PL intensity of 50 MoS_2_ flakes randomly collected on a molten glass substrate. An optical image (e), the corresponding PL map (the emission wavelength of 686.8 nm) (f) and the Raman map (A_1g_ peak at 403.1 cm^−1^) (g) of the as-grown MoS_2_ flakes.

To investigate the effects of the metal source feeding process in the DP method, we first studied the uniformity of as-grown MoS_2_ over a 2.5 cm × 1.0 cm substrate. A movie (Movie S1) shows the distribution of flakes on the whole substrate, indicating a highly uniform distribution. We analyzed the nuclei density (Fig. 2a), average area (Fig. 2b) and perimeter (Fig. S10) of each image extracted from the movie, and found that all show narrow distributions. The average nucleation density is 1080 flakes/mm^2^, the average perimeter of individual MoS_2_ flake is 47 μm, and the average area is 92 μm^2^. These values are highly uniform for 140 images over 2.5 cm × 1.0 cm area (Fig. S11). The above results have demonstrated that MoS_2_ grown by the DP method is highly uniform over a centimeter-scale substrate.

We then studied the quality and uniformity of the DP-grown TMDC flakes. First, we randomly chose 50 flakes from a molten glass substrate and analyzed their E_2g_ and A_1g_ Raman peaks (Fig. 2c). All the MoS_2_ flakes had an E_2g_ peak in the range 384.0 to 385.5 cm^−1^, and an A_1g_ peak in the range 402 to 404 cm^−1^, with the frequency differences between E_2g_ and A_1g_ peaks in a narrow range of 17.6–18.6 cm^−1^. Second, we measured photoluminescence (PL) spectra of the same 50 flakes and found that most of A exciton peaks of MoS_2_ were distributed from 684 to 689 nm (Fig. 2d). Both results indicate the uniformity of the DP-grown TMDCs. Third, we investigated an individual MoS_2_ flake. The PL intensity map at 686.8 nm (Fig. 2f) and Raman intensity map in the A_1g_ mode at 403.1 cm^−1^ (Fig. 2g) for the area shown in Fig. 2e show a quite uniform intensity over the whole flake. We noticed that for an individual MoS_2_ flake on molten glass, (i) the Raman spectrum showed an E_2g_ at 385.2 cm^−1^ and an A_1g_ at 402.8 cm^−1^ with a difference of 17.6 cm^−1^, smaller than that of monolayer MoS_2_ grown on a SiO_2_/Si substrate, (ii) the A exciton peak located at 686.8 nm showed a red shift compared with MoS_2_ grown on a SiO_2_/Si substrate. These changes may be ascribed to a strain effect and dielectric screening between the monolayer MoS_2_ and molten glass [33–35], since after the flake was transferred onto a SiO_2_/Si substrate, the Raman peaks and A exciton of DP-grown MoS_2_ are similar to those of exfoliated monolayer ones (Figs S12 and S13). To sum up, the above results indicate that the MoS_2_ grown using the DP method is a uniform monolayer.

**Figure 3.**
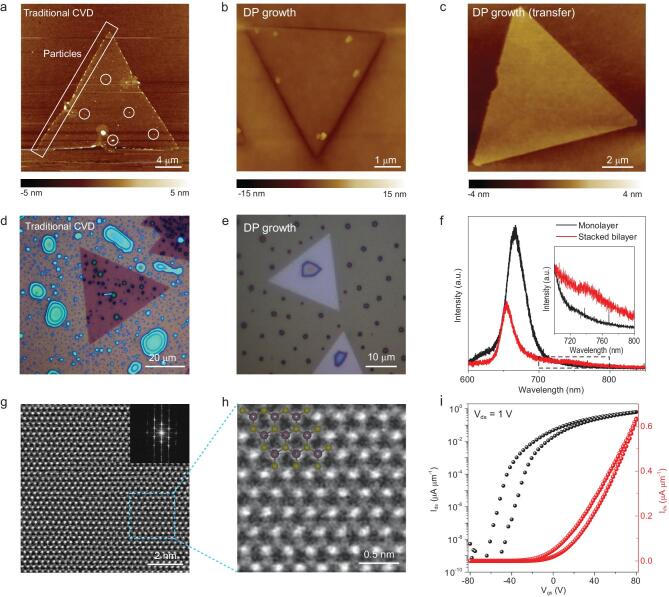
DP-grown MoS_2_ with a clean surface and high quality. (a) AFM image of traditional CVD-grown MoS_2_ with lots of particles absorbed at the edge and on the plane. (b) AFM image of DP-grown MoS_2_ with a clean surface. (c) High resolution AFM image of DP-grown MoS_2_ transferred onto a SiO_2_/Si wafer. Optical images of (d) traditional CVD-grown MoS_2_ and (e) DP-grown MoS_2_ after treatment with moisture TiCl_4_ vapor. (f) PL intensity profile of as-grown monolayer MoS_2_ and artificially stacked bilayer MoS_2._ (g) STEM image of DP-grown MoS_2_. The inset shows the corresponding FFT pattern. (h) Enlarged STEM image of the blue area denoted in (g). The inset shows the structural model of MoS_2_. (i) Transfer curves of a field effect transistor made of the DP-grown monolayer MoS_2_.

We then investigated the surface cleanness of the DP-grown MoS_2_ flakes. First, AFM was used to characterize the surface flatness. For MoS_2_ flakes grown using the traditional CVD with solid MoO_3_ powder as the metal source, many small particles were observed at the edges as well as on the plane (Fig. 3a). This feature is reported in the literatures using similar methods [5,13,36,37]. In sharp contrast in Fig. 3b, the MoS_2_ flakes grown using the DP method exhibit a clean surface except for a protrusion under each flake which is the Mo precursor confirmed by the AFM images of transferred MoS_2_ in Fig. 3c. Second, we exposed the surface of the substrate on which the MoS_2_ flakes had grown to TiCl_4_ vapor in humid air. Since TiCl_4_ is easily hydrolyzed to form TiO_2_ particles, which will be selectively absorbed on contaminated area of MoS_2_ [38]. As shown in Fig. 3d and e, the flakes grown by traditional CVD had many TiO_2_ particles while the DP-grown flakes showed only a few. These observations also show that the DP-grown MoS_2_ are much cleaner than traditional CVD-grown ones. Third, we checked the interlayer coupling of two monolayer MoS_2_ stacked structures. Generally, two stacked MoS_2_ layers with a clean interface will have a strong interlayer coupling characterized by suppressed monolayer PL emissions and the appearance of interlayer optical transitions [39,40]. We observed both a clear suppression of the A exciton emissions at ∼660 nm and the emergence of an interlayer emission peak at around 740 nm in the stacked bilayer MoS_2_ grown by the DP method (Fig. 3f), which indicates a clean surface of the monolayers. This phenomenon is in striking contrast to what was observed when stacking layers grown by the traditional CVD (Fig. S14), where no interlayer coupling was observed. The clean surface grown by the DP method originated from the reaction between the surface-limited diffusion of the Mo source and the S vapor which is supplied in a different gas-phase path, therefore secondary nucleation process is prohibited.

We also characterized the quality and electrical performance of the DP-grown MoS_2_. Scanning-transmission electron microscopy (STEM) images show the DP-grown MoS_2_ maintain perfect hexagonal lattice without apparent defects. The corresponding fast Fourier transform (FFT) pattern confirms the 2H phase of MoS_2_ (Fig. 3g and h). The XPS results for the DP-grown MoS_2_ show the typical binding energies of Mo 3d_5/2_ (229.5 eV), Mo 3d_3/2_ (232.7 eV), S 2p_3/2_ (162.3 eV), and S 2p_1/2_ (163.6 eV), with a Mo:S atomic ratio of 1:1.97 (Fig. S15). We also fabricated several field-effect transistors using the DP-grown MoS_2_ (Figs 3i and S16–17), which showed a decent carrier mobility in range of 7.5–21.5 cm^2^ V^ −1^ s^−1^ and an on/off ratio in range of 10^6^–10^8^. These results confirm the high quality of the DP-grown MoS_2_ flakes which are comparable to other growth techniques.

**Figure 4.**
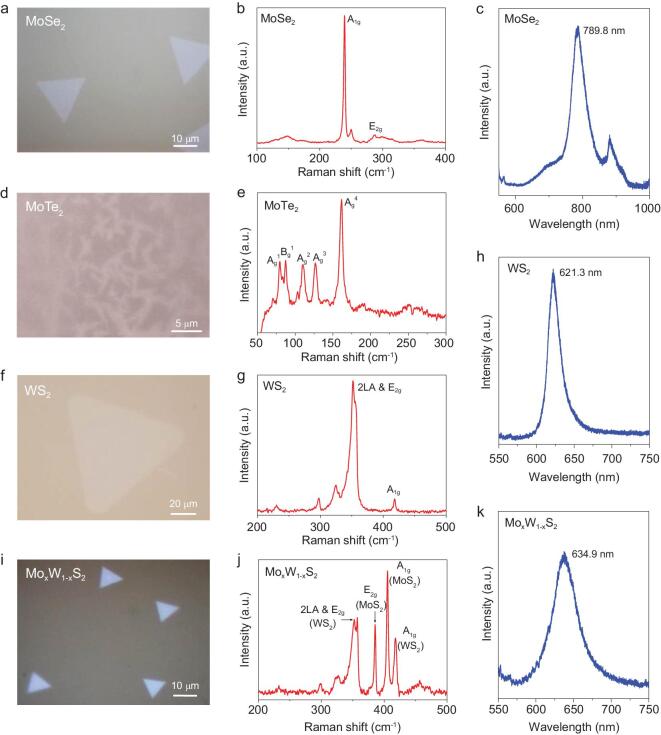
Universality of the DP method to grow different TMDCs. Optical images and the corresponding Raman and PL spectra of DP-grown MoSe_2_ flakes (a–c), 1T phase MoTe_2_ flakes (d, e), WS_2_ flakes (f–h) and Mo_x_W_1-x_S_2_ alloy flakes (i–k).

We found that DP is a universal method to grow various TMDCs besides MoS_2_, as well as to grow TMDC alloys and doped TMDCs. As shown in Fig. 4a, DP-grown MoSe_2_ flakes have a typical triangular shape with a size ranging from 10 to 25 μm. Figure 4b shows the two characteristic peaks (A_1g_ and E_2g_) of MoSe_2_ at 239.6 and 287.2 cm^−1^, respectively. There is no peak at around 350 cm^−1^, indicating that the as-grown flakes are monolayer MoSe_2_. The PL spectrum (Fig. 4c) shows a direct bandgap peak at 789.8 nm [40,41]. This method also works well for MoTe_2_, which is not easy to grow by the traditional CVD method. Figure 4d and e shows the needle-like shape of 1T^′^ phase MoTe_2_ and its typical Raman spectrum, which agrees well with that of mechanically exfoliated 1T^′^ MoTe_2_ [42]. We have also extended this DP method to grow monolayer WS_2_ (Fig. 4f). An E_2g_ peak of WS_2_ at 358.6 cm^−1^ and an A_1g_ peak at 418.7 cm^−1^ are observed (Fig. 4g). The frequency difference between these two modes is about 60.1 cm^−1^ and the PL peak position is located at 621.3 nm (Fig. 4h), good matches with the values for monolayer WS_2_ [43]. The above results demonstrate the versatility of the DP method in growing various TMDCs.

Furthermore, the DP growth method can be used to grow Mo_x_W_1-x_S_2_ alloy and V-doped MoS_2_. An optical image of Mo_x_W_1-x_S_2_ is shown in Fig. 4i. The Raman spectrum contains characteristic peaks of both WS_2_ and MoS_2,_ where the E_2g_ peak and A_1g_ peak of MoS_2_ are located at 385.8 and

404.8 cm^−1^, respectively, and those of WS_2_ are located at 357.4 and 418.3 cm^−1^, respectively (Fig. 4j). The PL peak of the Mo_x_W_1-x_S_2_ alloy is located at 639.4 nm as shown in Fig. 4k, which is between the wavelengths of pristine MoS_2_ and WS_2_ [44]. Recent study shows that a V-doped WSe_2_ monolayer is a room temperature ferromagnetic semiconductor [45], and the DP method can be used to grow such flakes. As shown in Fig. S18, both low and high concentration V-doped MoS_2_ monolayers were grown. The effective doping of V was further verified by the XPS results shown in Fig. S19. Taken together, these results have demonstrated that the DP method is a general approach to grow a family of TMDCs.

## CONCLUSION

We have developed a DP method for TMDC growth. In this method, the metal source is provided by diffusion through a thin molten glass substrate, leading to a uniform distribution of metal precursor and restricting the growth to only a surface reaction. As a result, highly uniform and monolayer TMDCs with clean surfaces have been grown on centimeter-scale glass substrates. We have also extended the method to the growth of other TMDCs to demonstrate its universality. These results highlight a new general approach for the growth of uniform and clean 2D materials for widespread applications.

## METHODS

### Embedding the metal precursor inside a glass substrate

First, a Na_2_MoO_4_ (0.94 mg) solution (4 μL, 1 mol L^−1^ in DI water) was dropped onto a 2-mm-thick soda lime glass slide with a size of 1.0 cm × 1.0 cm or 1.0 cm × 2.5 cm, and then dried in an oven. Then, a thinner (0.15-mm thick) soda lime glass slide of the same size was then placed on top of the above thick glass and the two were heated in a muffle furnace at 10°C min^−1^ to 660°C where they were sintered for 30 min to join them with the precursors remaining in between. For other studies the Na_2_MoO_4_ was replaced with Na_2_WO_4_ or a mixture of Na_2_MoO_4_ and Na_2_WO_4_ or a mixture of Na_2_MoO_4_ and NaVO_3_. The above fused glass sandwich was used as both the growth substrate and metal source for the DP growth. Note that the fused glass with the metal precursor inside is named SG-M, where M is Mo or W or a mixture.

### DP growth of MoS_2_

A horizontal tube furnace was used for the DP growth of MoS_2_. S powder (150 mg, 99.5%, Sigma-Aldrich) was loaded upstream where the temperature was 150°C and the SG-Mo was placed in the center of the furnace, serving as Mo precursor and growth substrate simultaneously. Before growth, the tube was pumped to 0.05 Torr and refilled with Ar to ambient pressure which was repeated three times to eliminate residual oxygen and water. During the growth, the temperature was increased to 730–750°C at a rate of 50°C min^−1^ and the growth lasted for 10–20 min. Ar was used as the carrier gas with a flow rate of 80 sccm at 1.2–2.0 Torr. After growth, the furnace was cooled to 200°C under 80 sccm of Ar flow.

### DP growth of other TMDCs and alloys

For the DP growth of MoSe_2_, MoTe_2_, WS_2_, Mo_x_W_1-x_S_2_ and V-doped MoS_2_, we followed a similar growth procedure to that used for MoS_2_ but with slight modifications. **(i) MoSe_2_ growth:** Se powder (200 mg, 99.5%, Sigma-Aldrich) was loaded in the furnace position where the temperature was 280°C. The growth temperature was 750–800°C. The 80 sccm Ar and 8 sccm H_2_ were used as the carrier gas at a low pressure of 1.2–2.0 Torr. **(ii) MoTe_2_ growth:** Te powder (200 mg, 99.5%, Sigma-Aldrich) was loaded upstream about 11 cm away from the center of the furnace. The growth temperature was 730–750°C. The 120 sccm Ar and 20 sccm H_2_ were used as the carrier gas at ambient pressure. **(iii) WS_2_ growth:** S powder (150 mg, 99.5%, Sigma-Aldrich) was loaded upstream where the temperature was 190°C, and SG-W was placed in the center of the furnace. The growth temperature was 730–780°C. The 80 sccm Ar and 4 sccm H_2_ were used as the carrier gas at ambient pressure. **(iv) Mo_x_W_1-x_S_2_ growth** S powder (150 mg, 99.5%, Sigma-Aldrich) was loaded upstream where the temperature was 150°C. SG-Mo-W (the ratio of Na_2_MoO_4_:Na_2_WO_4_ was 1:15) was placed in the center of the furnace. The growth temperature was 750–780°C. The 80 sccm Ar and 4 sccm H_2_ were used as the carrier gas at a low pressure of 1.2–2.0 Torr. **(v) V-doped MoS_2_ growth:** S powder (120 mg, 99.5%, Sigma-Aldrich) was loaded upstream where the temperature was 150°C. SG-Mo-V (for low concentration V doping, the ratio of Na_2_MoO_4_:NaVO_4_ was 1:1, for high concentration V doping, the ratio of Na_2_MoO_4_:NaVO_4_ was 1:5) was placed in the center of the furnace. The growth temperature was 730–750°C. The 120 sccm Ar and 8 sccm H_2_ were used as the carrier gas at a low pressure of 1.2–2.0 Torr.

### Growth of MoS_2_ by traditional CVD

A horizontal tube furnace was used for the growth of MoS_2_. S powder (100 mg, 99.5%, Sigma-Aldrich) was loaded upstream at 220°C. MoO_3_ powder (10 mg, 99.5%, Sigma-Aldrich) was placed in the center of the furnace. SiO_2_/Si (Si substrate with a 300 nm thick thermally grown oxide) was used as growth substrate and placed on top of MoO_3_ powder with the oxide layer facing down. The temperature was increased to the growth temperature of 700°C at a rate of 50°C min^−1^. During growth, Ar was used as the carrier gas with a flow rate of 80 sccm at ambient pressure, and the growth lasted for 10 min.

### Transfer of TMDCs

A polyethylene terephthalate (PET) film was placed on the obtained TMDC/glass and its temperature was increased to 65°C. When the PET had softened, the TMDC became attached to PET to form PET/TMDC/glass. The removal of PET/TMDC from the glass substrate is through a very slow mechanical peeling followed by placing on a target substrate and heated to above 90°C to cause the PET/TMDC to stick tightly to the target substrate. The PET/TMDC/target substrate was then immersed in dichloromethane to get rid of the PET, leaving the TMDC on the target substrate. Here, the target substrates could be either SiO_2_/Si or TEM grids.

### Material characterization

The morphology and surface of the samples were examined by an optical microscope (Carl Zeiss Microscopy, Germany), SEM (5 kV, Hitachi SU8010, Japan) and AFM (Cypher ES, Asylum Research, USA). Raman and PL spectra were collected using 532 nm laser excitation with a beam size of ∼1 μm (Horiba LabRAB HR Evolution Japan). Chemical elemental analyses of the samples were conducted by XPS (monochromatic Al Kα X-rays, 1486.6 eV, PHI VersaProbe II, Japan). HAADF-STEM images were taken by an aberration-corrected TEM (FEI Titan Cube Themis G2 with a field emission gun at 60 kV, USA), with a resolution of 0.8 Å. The acquisition parameters were set as below, i.e. probe size of 9, condenser lens aperture of 50 μm, and camera length of 145 mm.

### Device fabrication and measurements

FET devices were fabricated using a laser writing system (Aresis Dell, ZKS). In brief, a drop of AZ5214 photoresist (PR) was spin-coated onto the SiO_2_/Si substrate with the samples attached (2000 rpm for 1 min), and baked at 125°C for 1 min. Photo-lithography was then conducted using PR as a positive resist. This was followed by successive develop, metal deposition, and lift-off to fabricate the FET devices. The metal electrodes were made of 5 nm Cr and 50 nm Au, which were deposited using e-beam evaporation. The device measurements were performed in a vacuum probe station (10^−5^ mBar, Lakeshore TTPX, USA).

## Supplementary Material

nwaa115_Supplemental_Files
