# Supplementary material for: Dissolution-precipitation growth of uniform and clean two dimensional transition metal dichalcogenides
Source: Natl Sci Rev. 2020 May 30;8(3):nwaa115. doi: 10.1093/nsr/nwaa115 (PMC8288458; doi:10.1093/nsr/nwaa115)
Supplement: nwaa115_Supplemental_Files [file nwaa115_supplemental_files.zip › Supplementary data.docx]

Supplementary Information for ***National Science Review***

**Dissolution-Precipitation Growth of Uniform and Clean Two Dimensional Transition Metal Dichalcogenides**

Zhengyang Cai^1,†^, Yongjue Lai^1, †^, Shilong Zhao^1^, Rongjie Zhang^1^, Junyang Tan^1^, Simin Feng^1^, Jingyun Zou^1^, Lei Tang^1^, Junhao Lin^3^, Bilu Liu^1,*^, Hui-Ming Cheng^1,2,*^

^1^Shenzhen Geim Graphene Center, Tsinghua−Berkeley Shenzhen Institute and Tsinghua Shenzhen International Graduate School, Tsinghua University, Shenzhen 518055, China

^2^Shenyang National Laboratory for Materials Sciences, Institute of Metal Research, Chinese Academy of Sciences, Shenyang 110016, China

^3^Department of Physics, Southern University of Science and Technology, Shenzhen 518055, China

^†^These authors contribute equally.

^*^Corresponding authors:

[bilu.liu@sz.tsinghua.edu.cn](mailto:bilu.liu@sz.tsinghua.edu.cn) (BL);

[hmcheng@sz.tsinghua.edu.cn](mailto:hmcheng@sz.tsinghua.edu.cn) (HMC)

**Supplementary Figure S1.** The molten status of two pieces of glass after heat treatment at various temperatures of (a) 600 °C, (b) 750 °C and (c) 850 °C. At 600 °C, the glasses do not melt and they can be separated easily. At 750 °C, the glasses fuse together and edges become soft, indicating this temperature is suitable for growth. At 850 °C, the molten glasses form a round shape showing the temperature is too high. As a result, a temperature around 750 °C was chosen for the DP growth of TMDCs.

**Supplementary Figure S2.** Cross-sectional optical images of the glass substrate after DP growth. (a) Low magnification view of the junction area of the two glass pieces. (b) Magnified view of the junction area. (c) Optical image of an area just below the junction. The bright line in the center (b) is the junction between the two pieces of glass, and the thickness of top piece of glass is 0.14 mm (a), slightly smaller than the original (0.15 mm) due to melting and shrinkage. Below the junction in (c), a few small particles are observed, while no flakes are observed (c), indicating that no Mo source has escaped from the edge areas.

**Supplementary Figure S3.** The depth dependent morphology of the glass substrate using the DP growth. (a) Photograph of the Na_2_MoO_4_/glass substrate with a size of 1.0 cm x 1.0 cm before and after growth. The white circle is the sandwiched Na_2_MoO_4_ precursor. (b) Schematic showing the method to change the focus of an optical microscope to observe the flakes grown on the surface and the interlayer area of the Mo precursor. (c-d) Optical images of (c) MoS_2_ grown on the surface, and (d) the Mo species at the interlayer of the pieces of glass. By changing the focus of the microscope, we can observe the MoS_2_ flakes on the surface are typically triangles, while the interlayer material consists of thick, irregular shaped Mo source. The results demonstrate that Mo source diffuses through the top piece of glass, which has a control of the total amount of metal precursor available for MoS_2_ growth.

**Supplementary Figure S4.** SEM images of the top of the glass substrate after DP growth. (a) A typical SEM image after DP growth. (b) A magnified image. (c) A typical SEM image showing four protrusions, and (d) Large magnification of a single protrusion with a lateral size of around 1-2 μm. These protrusions are formed by the diffusion of sandwiched Na_2_MoO_4_ precursor.


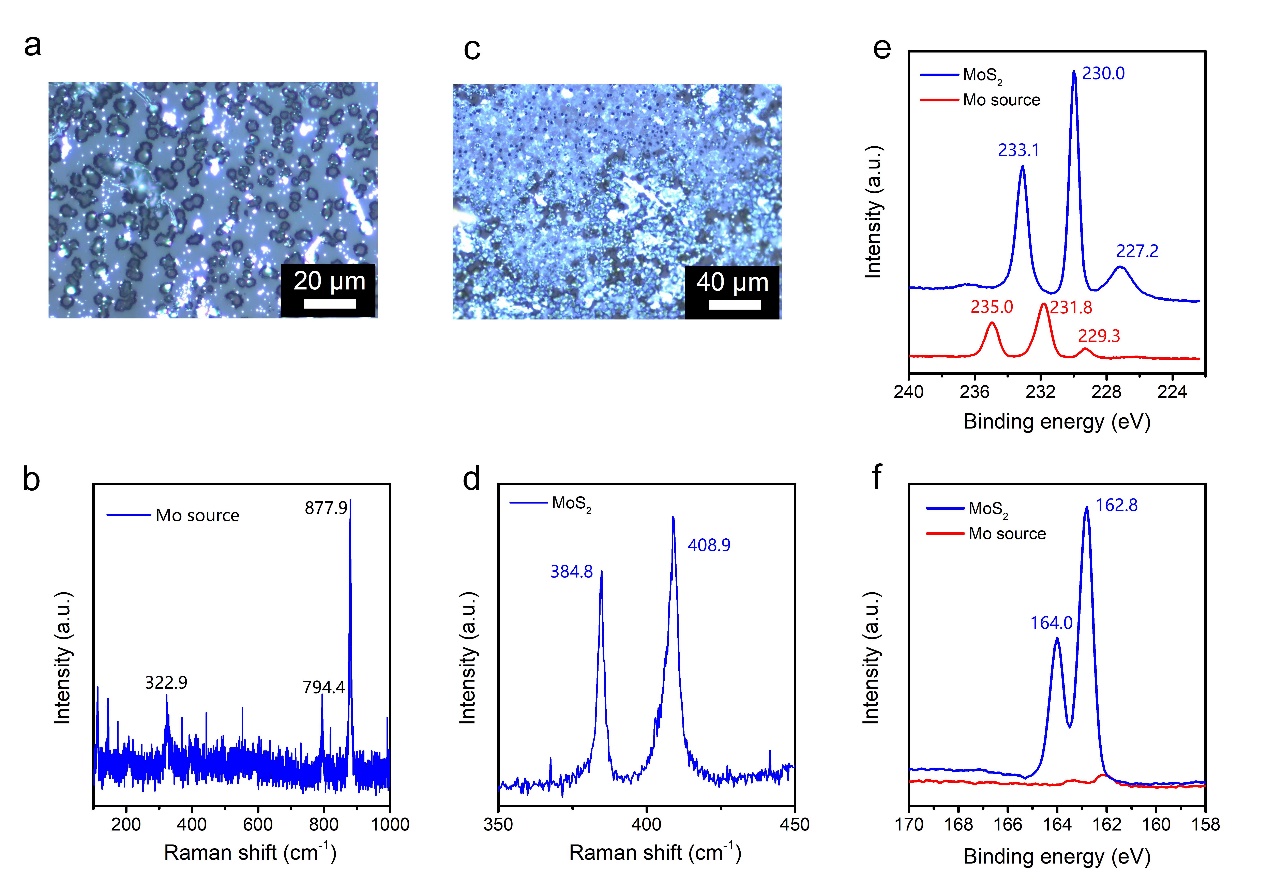


**Supplementary Figure S5.** DP growth of MoS_2_ using MoO_3_ as the precursor in a control experiment. (a) Optical image of the surface of the glass substrate after Mo source has diffused from the interlayer, and (b) the corresponding Raman spectrum. (c) Optical image of glass substrate after MoS_2_ growth, and (d) corresponding Raman spectrum indicating the formation of thick MoS_2_ flakes. (e, f) High resolution XPS spectra of (e) Mo 3d peaks and (f) S 2p peaks for both the Mo source and the grown MoS_2_. For sample (a), the Raman main peaks at 322.9 cm^-1^, 794.4 cm^-1^, and 877.9 cm^-1^ can be assigned to the Mo-O vibration, indicating the diffusion of MoO_3_ from under the glass. The Mo 3d peaks located at 231.8 eV and 235.0 eV indicate the valence state of elemental Mo is 6, and no obvious S signal is observed. For sample (c), the Mo 3d peaks at 230.0 eV and 233.1 eV, together with the S 2p peaks at 162.8 eV and 164.0 eV, indicate the existence of MoS_2_ in the grown samples. For comparison, we used MoO_3_ in place of Na_2_MoO_4_ since it has a much higher vapor pressure than Na_2_MoO_4_ at the growth temperature leading to a larger amount of Mo diffusing to the surface, resulting in large protrusions and the growth of very thick MoS_2_ flakes.

**Supplementary Figure S6.** Control experiment following the DP growth recipe but without covering the Na_2_MoO_4_ precursor with a thin glass layer. The optical images show the junction area. In the high concentration area (left), very thin triangular flakes can be observed while in the low concentration area (right), very small particles are grown. These results demonstrate that the DP growth controls the Mo feed to the surface, which is a key factor for the uniform growth of 2D materials.

**Supplementary Figure S7.** Control experiments at various temperatures and with different precursors to prove the diffusion of Mo species to the top surface of glass substrate. Optical images of the top surface and the interface where (a-b) are for the heat treatment of two pieces of glass at 750 °C but without the addition of Na_2_MoO_4_ at the interface and S to the gas stream, (c-d) are for the heat treatment of two pieces of glass at 750 °C with a Na_2_MoO_4_ interlayer but with no S feed, and (e-f) are for the heat treatment of the glass at 660 °C with a Na_2_MoO_4_ interlayer but with no S feed. Taking these results together, we systematically studied the formation of protrusions on the molten glass surface by treating it at different conditions. It is seen that protrusions are present in (c-d) but not in (a-b), indicating that the protrusions are produced by the Na_2_MoO_4_ precursor and not by the molten glass substrate itself. We also examined the surface of a freshly fused glass substrate (e-f) for which we cannot observe any protrusions on the surface, showing that the Na_2_MoO_4_ precursor is not able to diffuse out at 660°C. Hence, it is reasonable to assume that the protrusions are mainly composed of Mo which has diffused through the top molten glass to its surface at the DP growth temperature of 750°C.

**Supplementary Figure S8.** Location-dependent growth of MoS_2_ in a traditional CVD process which uses MoO_3_ powder and S powder to grow MoS_2_. (a) the optical image and (b) schematic illustrating that the Mo concentration on substrate surface is location dependent, leading to non-uniform nuclei on the substrate.


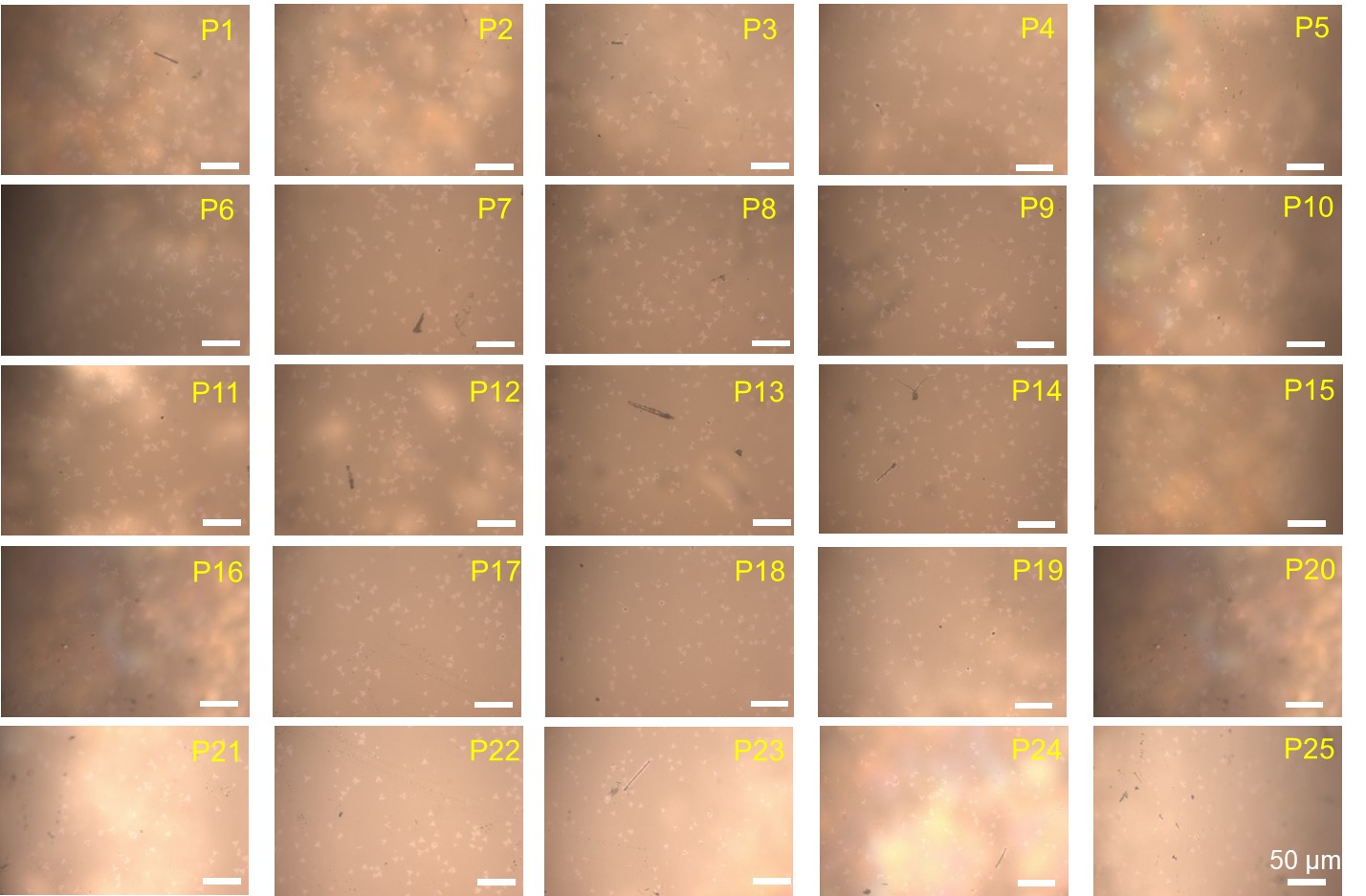


**Supplementary Figure S9.** Uniform distribution of DP grown MoS_2_ on a 1.0 cm x 1.0 cm glass substrate. These images show the highly uniform distribution of the as-grown MoS_2_ flakes.

**Supplementary Figure S10.** Statistical analysis of average perimeter of MoS_2_ flakes for 140 images taken on a 2.5 cm x 1.0 cm glass substrate.


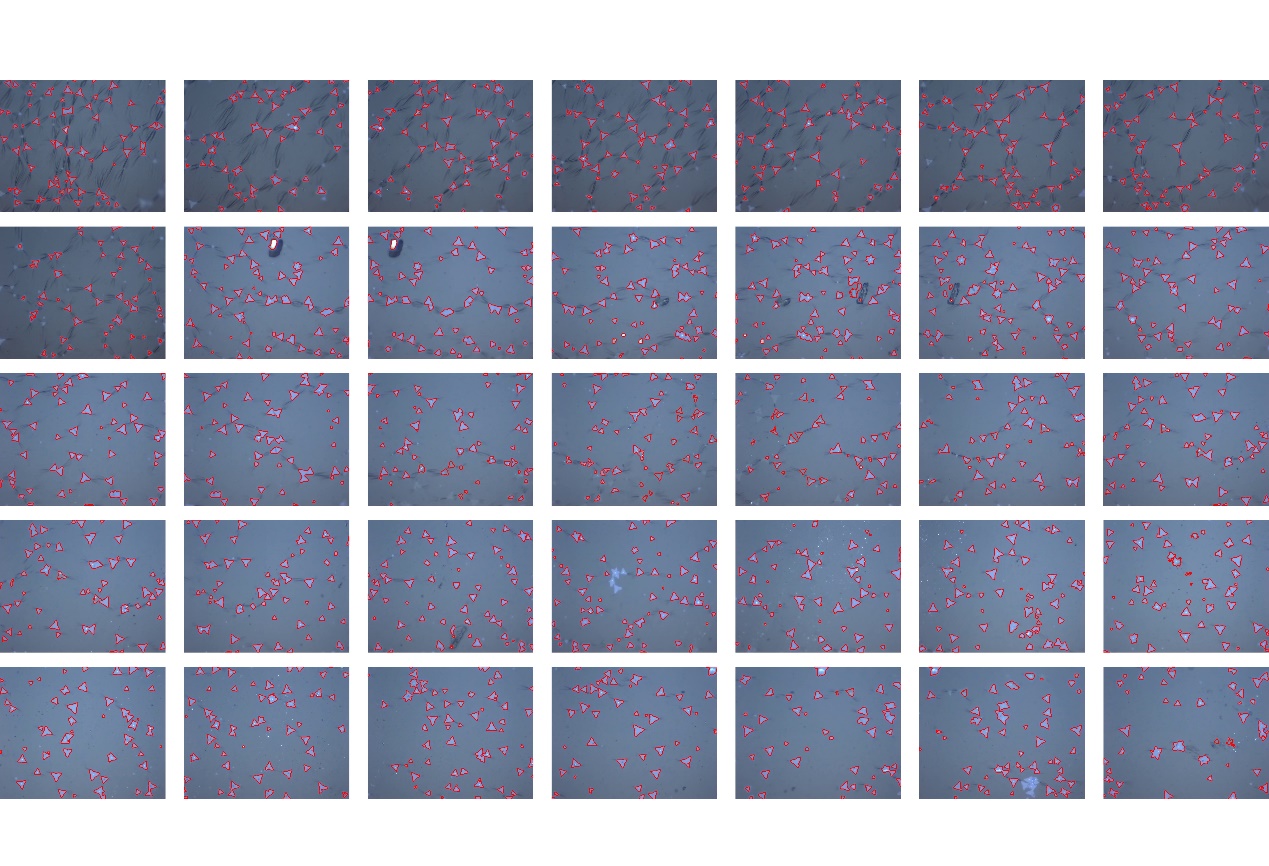

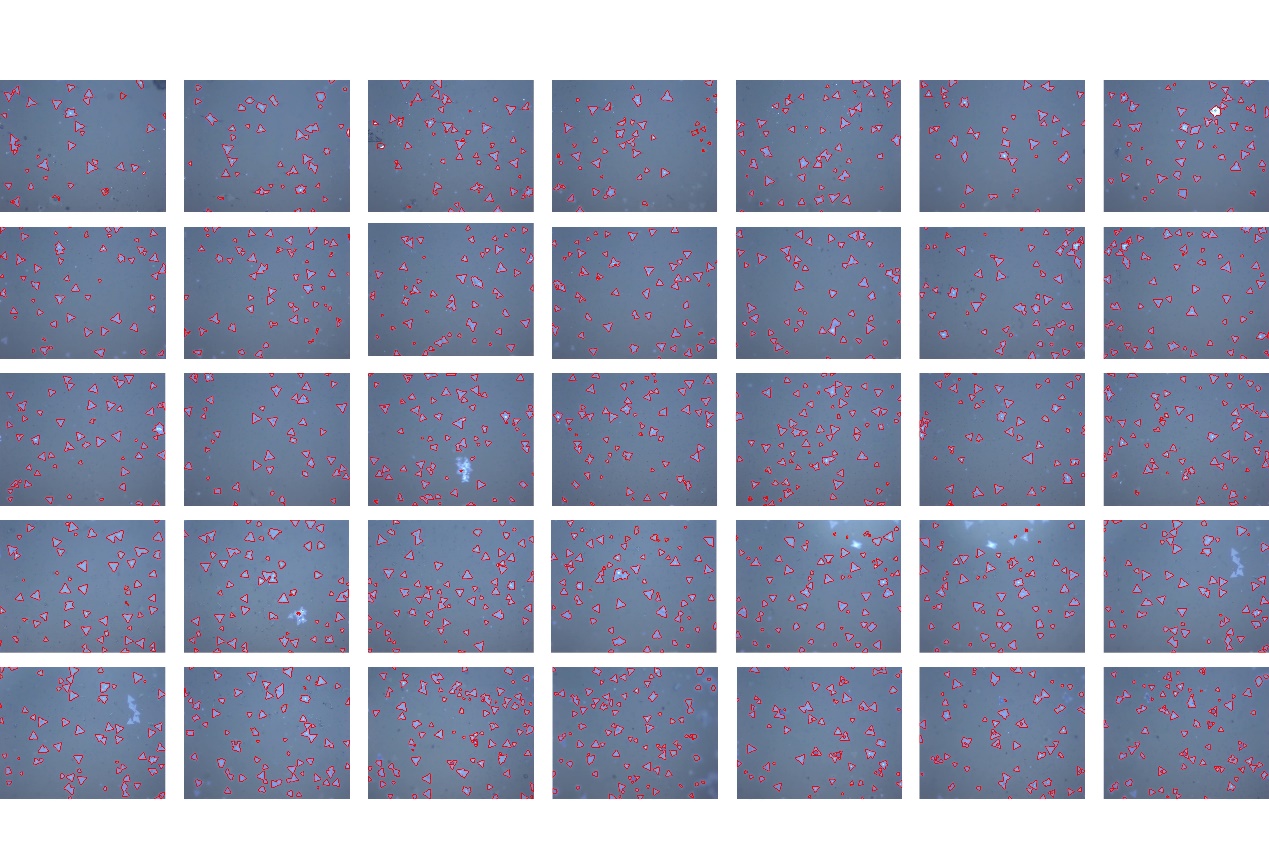


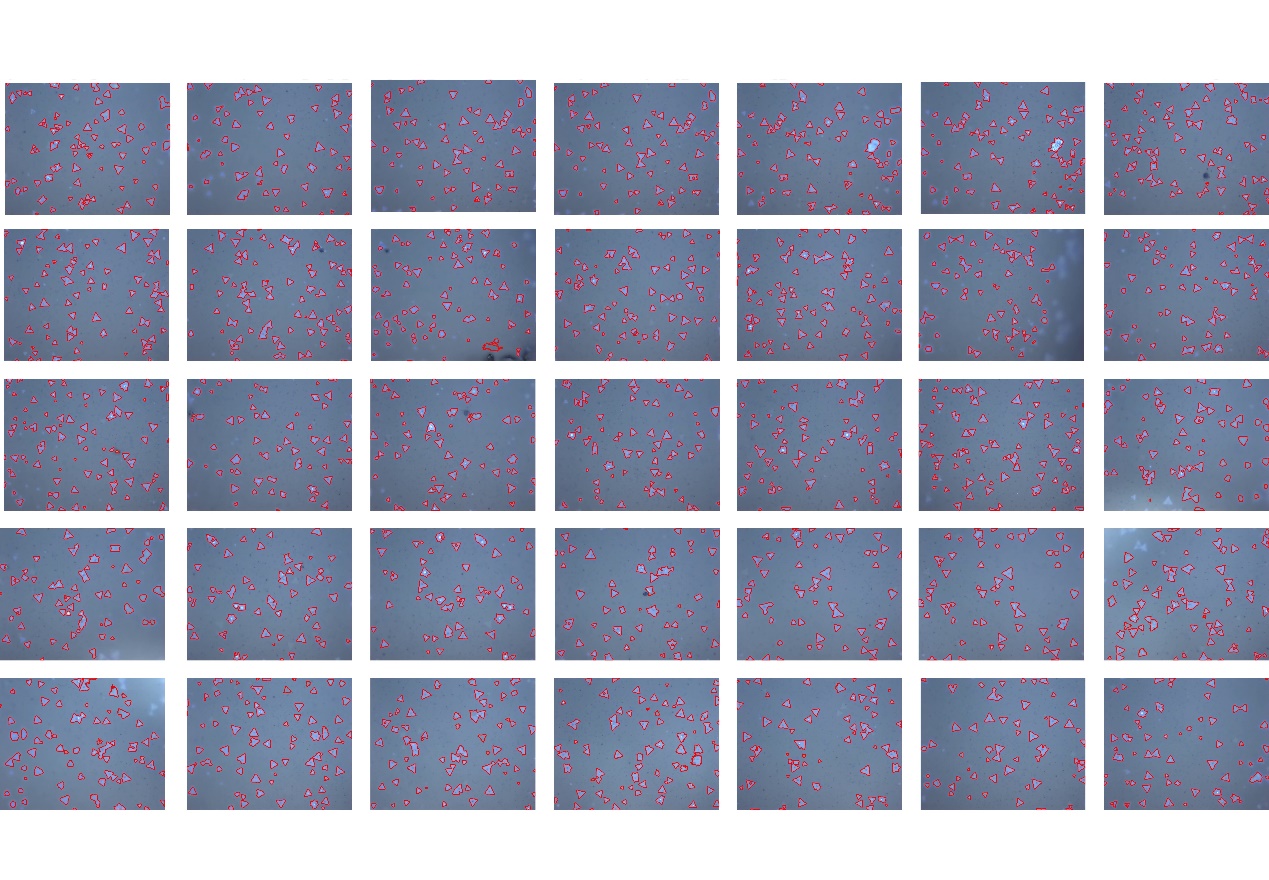


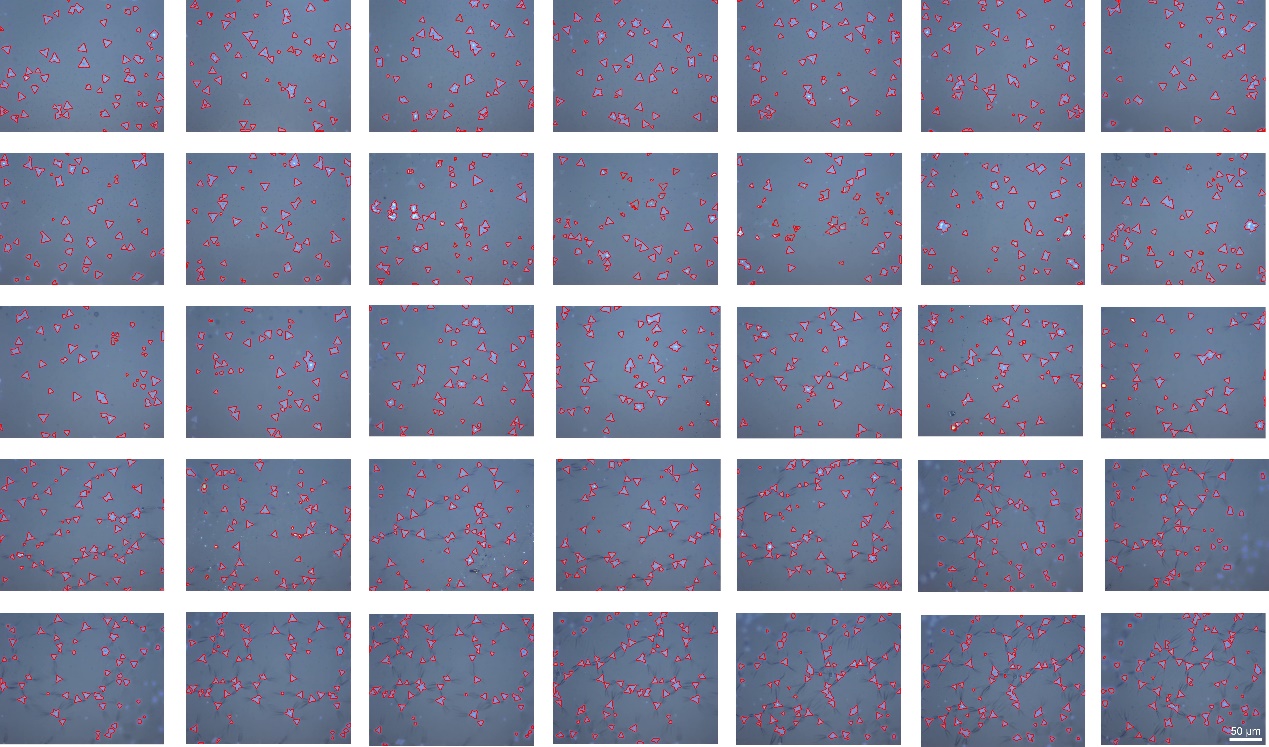


**Supplementary Figure S11.** Optical images of as-grown MoS_2_ flakes on 2.5 cm x 1.0 cm molten glass using the DP method. 140 images were collected and their contours have been marked to obtain their area and perimeter as shown in **Figures 2a and 2b**.

Treatment process using Python programming

1. From the **Movie S1**, more than 800 images were obtained each second. By excluding any blurred or repeated ones, 140 images were obtained.

2. Using the “OpenCV” package, gray transformation, threshold filter, and contrast strength tools were applied to the images to find the contours of the as-grown flakes for each image.

3. Based on the contours in each image, the nucleation density, perimeters, and coverages were calculated and statistically analyzed.


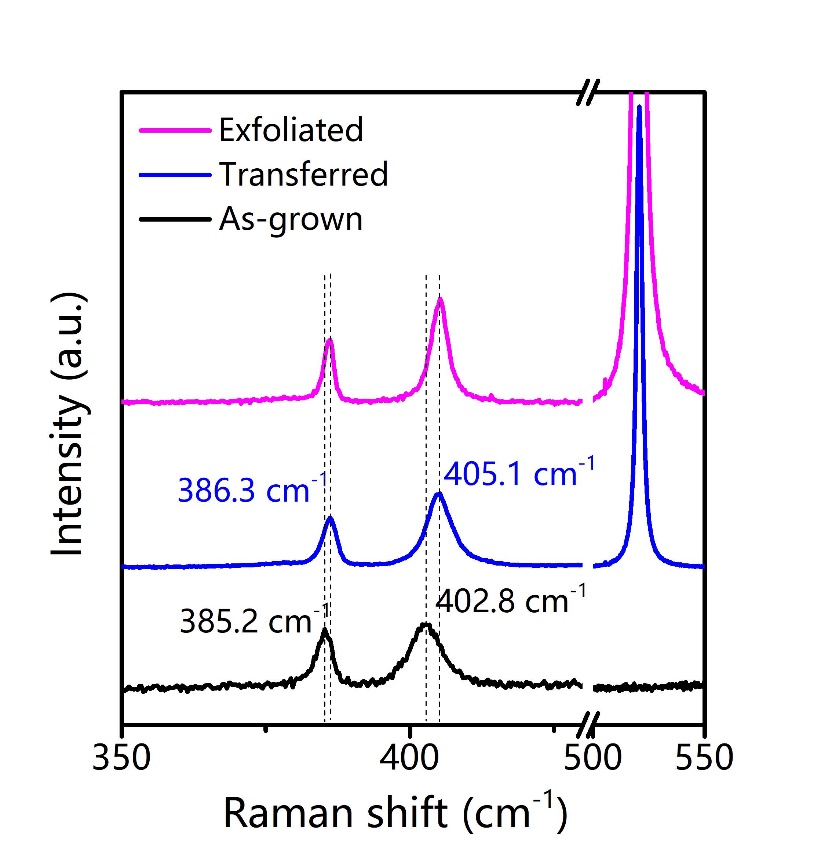


**Supplementary Figure S12.** Raman spectra of DP grown MoS_2_. Raman spectra of MoS_2_ grown on molten glass, transferred onto a SiO_2_/Si wafer and exfoliated MoS_2_ on SiO_2_/Si substrate. The peaks of the MoS_2_ grown on the molten glass are located at 385.2 cm^-1^ and 402.8 cm^-1^ for the E_2g_ and A_1g_ modes, respectively. The difference between the two peaks is 17.6 cm^-1^. After being transferred onto SiO_2_/Si, these two peaks shift to 386.3 cm^-1^ and 405.1 cm^-1^, respectively, and the difference is 18.8 cm^-1^. These values are the same as those of exfoliated MoS_2_ on SiO_2_/Si.


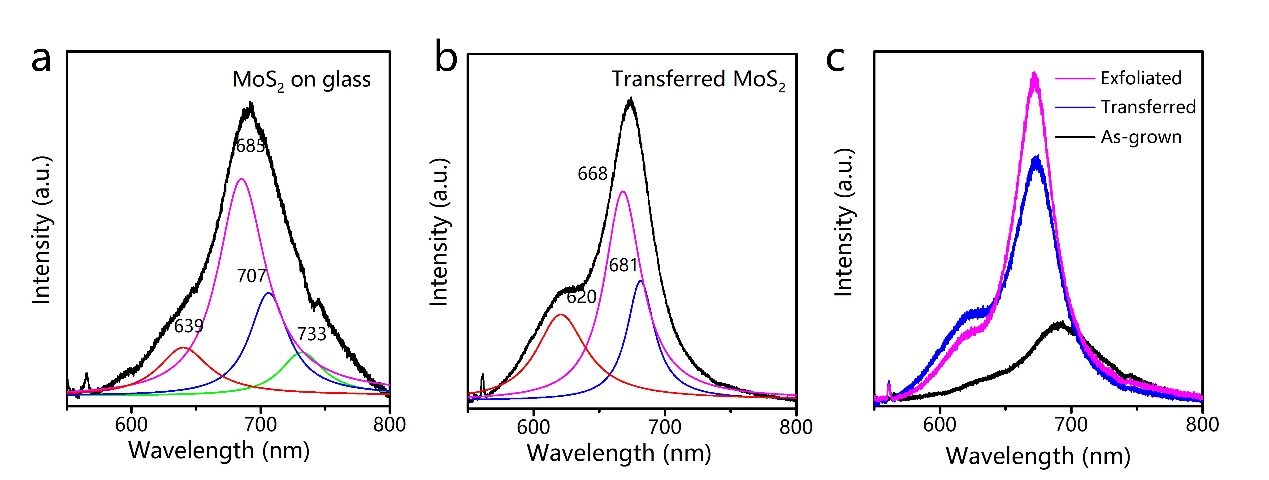


**Supplementary Figure S13.** PL spectra of MoS_2_ grown on molten glass and a sample transferred onto SiO_2_/Si. (a) The PL spectrum and fitted curves of MoS_2_ on molten glass. (b) The PL spectrum and fitted curves of MoS_2_ after transfer onto SiO_2_/Si. (c) A comparison of the PL spectra of MoS_2_ grown on molten glass, MoS_2_ transferred onto SiO_2_/Si, and exfoliated MoS_2_ on SiO_2_/Si. The results show that the different PL behavior of MoS_2_ grown on molten glass results from the effect of molten glass rather than the MoS_2_ itself.


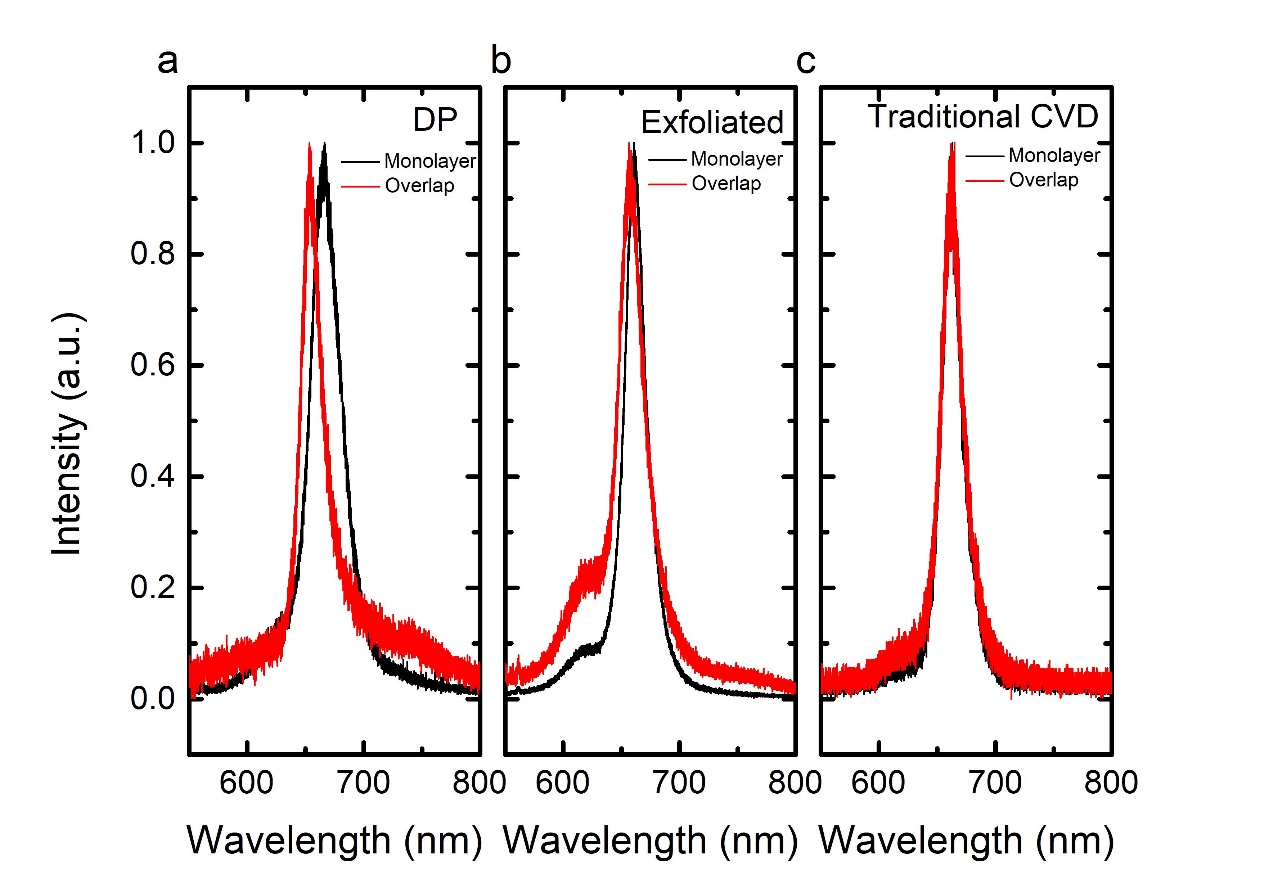


**Supplementary Figure S14.** PL spectra of monolayer MoS_2_ and artificially stacked bilayer MoS_2_ using three types of MoS_2_. (a) MoS_2_ grown by the DP method, (b) MoS_2_ exfoliated by the Scotch tape method, and (c) MoS_2_ grown by the traditional CVD method.


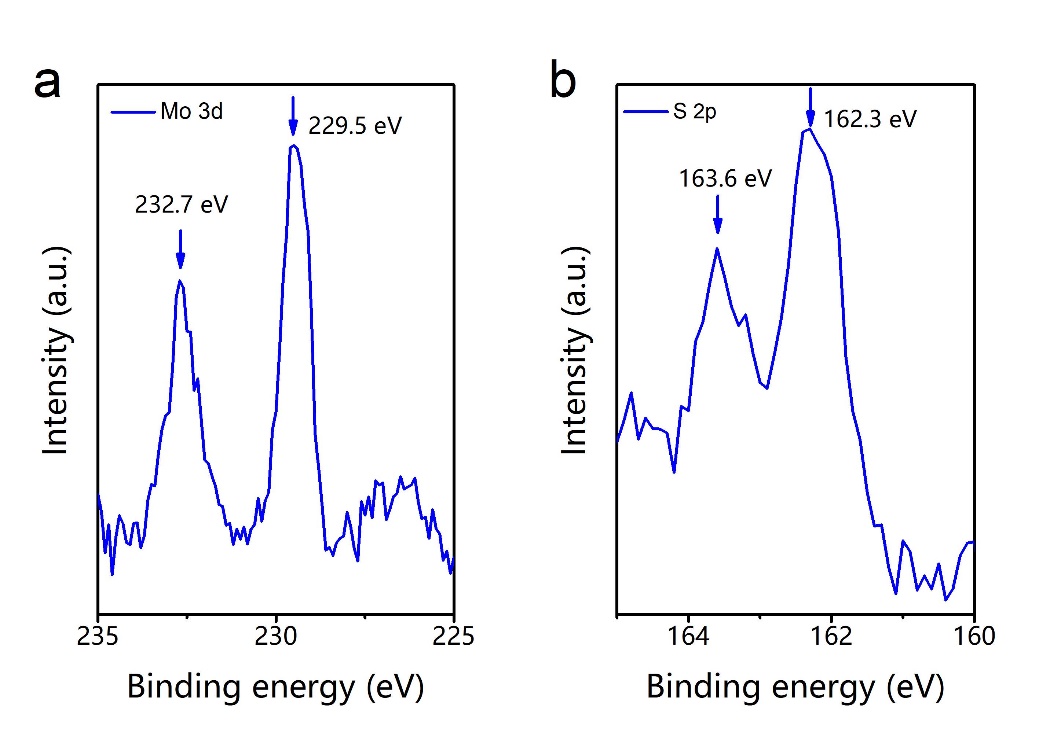


**Supplementary Figure S15.** XPS measurements of DP grown MoS_2_. (a) High resolution spectrum of the Mo 3d peaks. (b) High resolution spectrum of the S2p peaks. The atomic ratio of S and Mo contents is estimated to be 1.97, indicating the high quality of DP-grown MoS_2_.


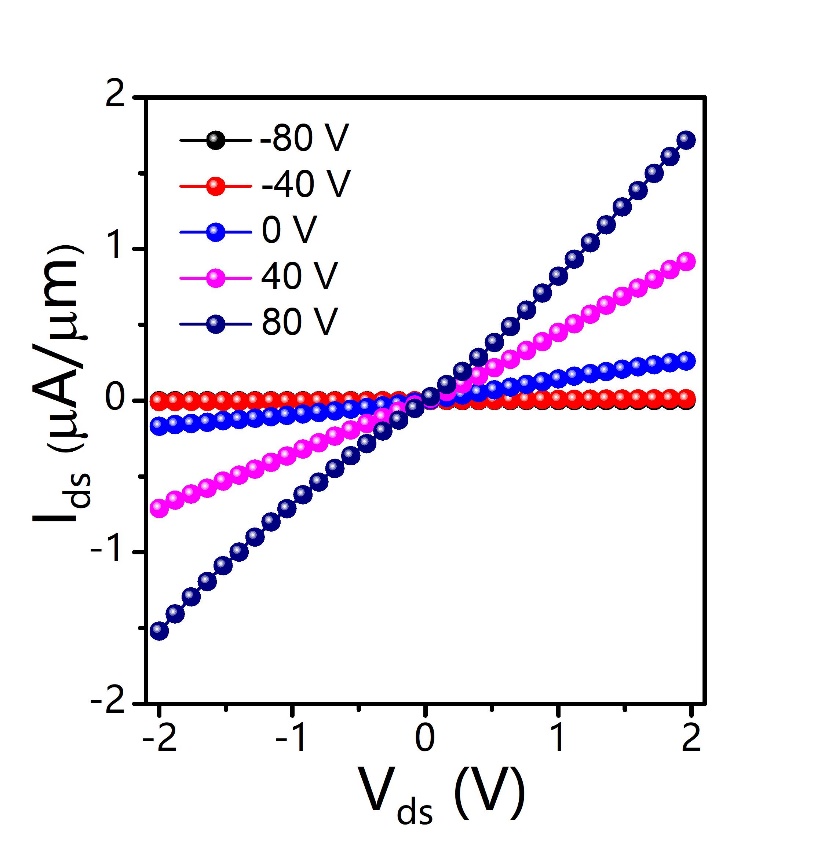


**Supplementary Figure S16.** Output curves of transferred MoS_2_ grown by the DP method.

**Supplementary Figure S17.** Additional FET devices made of DP-grown MoS_2_. (a-e) Optical images of five FET devices, corresponding transfer curves, and a summary of the channel length, width, and FET mobility of these devices.


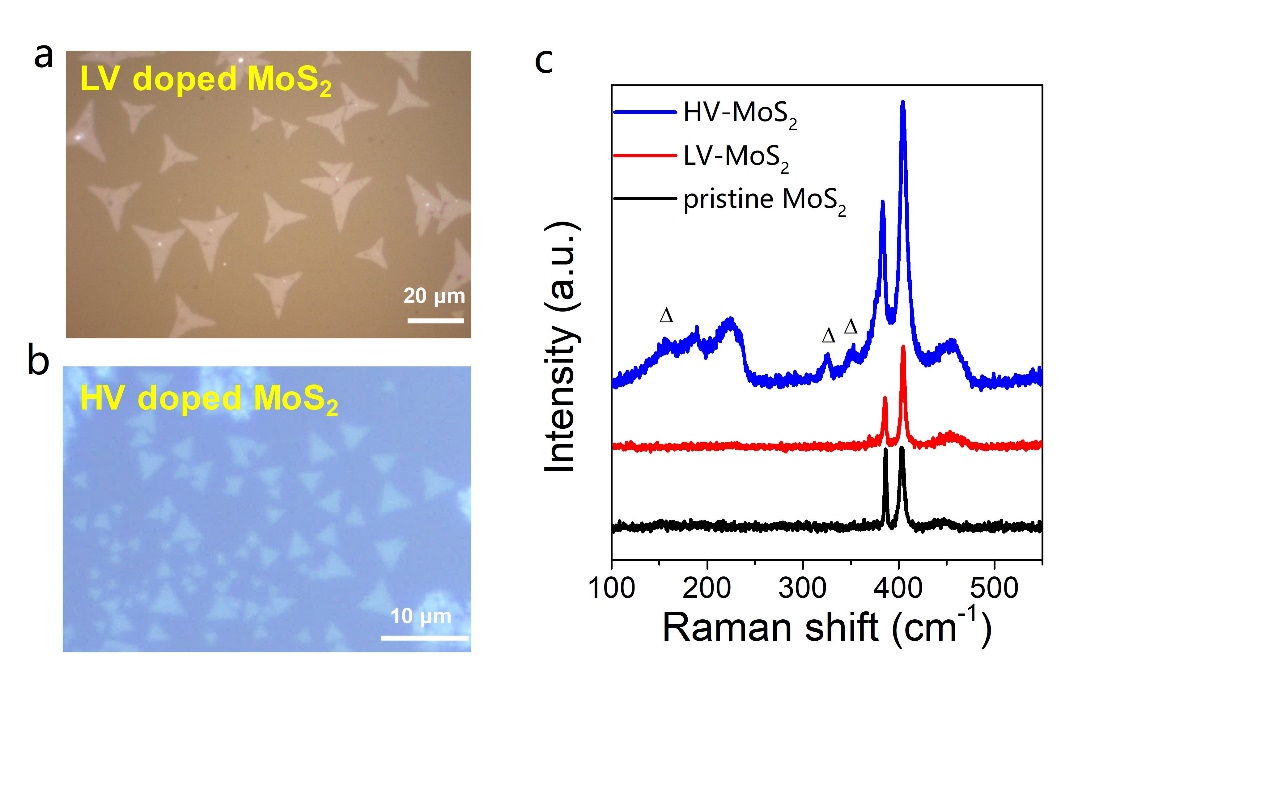


**Supplementary Figure S18.** Optical images and corresponding Raman spectra of (a, c) low concentration V-doped MoS_2_ flakes (LV-MoS_2_), and (b, d) high concentration V-doped MoS_2_ flakes (HV-MoS_2_).


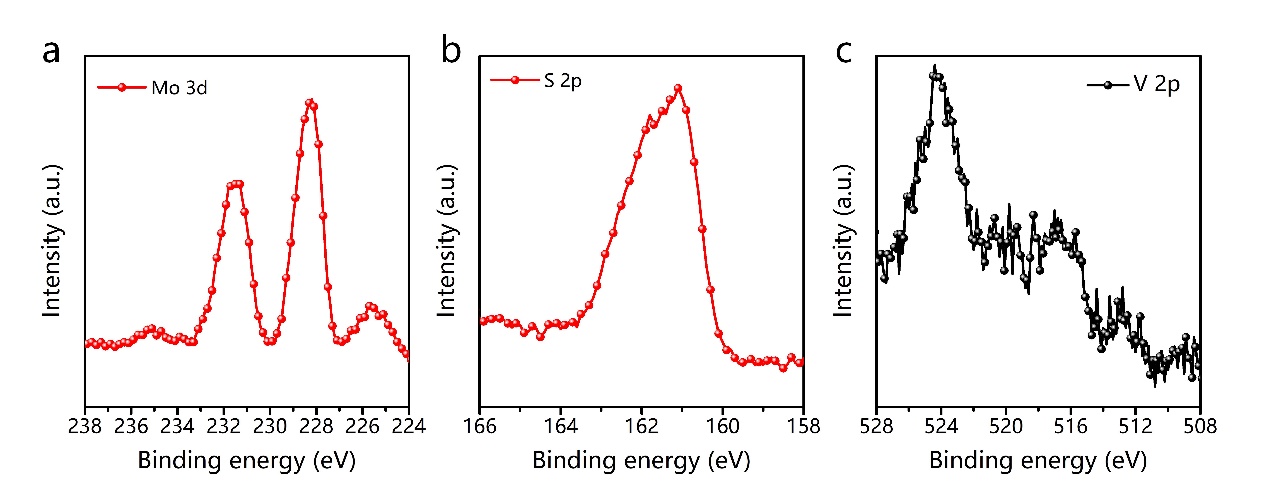


**Supplementary Figure S19.** XPS results of HV-MoS_2_ on a molten glass substrate. (a) High resolution curve of the Mo 3d peaks. (b) High resolution curve of the S2p peaks. (c) High resolution curve of the V2p peaks.
